# Single nucleotide polymorphism discovery in elite north american potato germplasm

**DOI:** 10.1186/1471-2164-12-302

**Published:** 2011-06-09

**Authors:** John P Hamilton, Candice N Hansey, Brett R Whitty, Kevin Stoffel, Alicia N Massa, Allen Van Deynze, Walter S De Jong, David S Douches, C Robin Buell

**Affiliations:** 1 Department of Plant Biology, Michigan State University, East Lansing MI, 48824, USA; 2 Seed Biotechnology Center, University of California, Davis, CA, 95616, USA; 3 Cornell University, Department of Plant Breeding & Genetics, Ithaca, NY, 14853, USA; 4 Department of Crop and Soil Sciences, Michigan State University, East Lansing MI, 48824, USA

## Abstract

**Background:**

Current breeding approaches in potato rely almost entirely on phenotypic evaluations; molecular markers, with the exception of a few linked to disease resistance traits, are not widely used. Large-scale sequence datasets generated primarily through Sanger Expressed Sequence Tag projects are available from a limited number of potato cultivars and access to next generation sequencing technologies permits rapid generation of sequence data for additional cultivars. When coupled with the advent of high throughput genotyping methods, an opportunity now exists for potato breeders to incorporate considerably more genotypic data into their decision-making.

**Results:**

To identify a large number of Single Nucleotide Polymorphisms (SNPs) in elite potato germplasm, we sequenced normalized cDNA prepared from three commercial potato cultivars: 'Atlantic', 'Premier Russet' and 'Snowden'. For each cultivar, we generated 2 Gb of sequence which was assembled into a representative transcriptome of ^~^28-29 Mb for each cultivar. Using the Maq SNP filter that filters read depth, density, and quality, 575,340 SNPs were identified within these three cultivars. In parallel, 2,358 SNPs were identified within existing Sanger sequences for three additional cultivars, 'Bintje', 'Kennebec', and 'Shepody'. Using a stringent set of filters in conjunction with the potato reference genome, we identified 69,011 high confidence SNPs from these six cultivars for use in genotyping with the Infinium platform. Ninety-six of these SNPs were used with a BeadXpress assay to assess allelic diversity in a germplasm panel of 248 lines; 82 of the SNPs proved sufficiently informative for subsequent analyses. Within diverse North American germplasm, the chip processing market class was most distinct, clearly separated from all other market classes. The round white and russet market classes both include fresh market and processing cultivars. Nevertheless, the russet and round white market classes are more distant from each other than processing are from fresh market types within these two groups.

**Conclusions:**

The genotype data generated in this study, albeit limited in number, has revealed distinct relationships among the market classes of potato. The SNPs identified in this study will enable high-throughput genotyping of germplasm and populations, which in turn will enable more efficient marker-assisted breeding efforts in potato.

## Background

The most widely cultivated potato species, *Solanum tuberosum *Group Tuberosum, is an autotetraploid (2n = 4x = 48) and the world's third most important food crop in overall production, after rice and wheat [1]. Potato improvement is constrained by numerous challenges and bottlenecks [2-5] including a high level of heterozygosity, tetraploid genetics, restricted genetic base, biotic and abiotic constraints as well as the need to simultaneously select for market-based quality traits and agronomic performance. While genetic maps and markers have been described in potato [6-9], they have not yet had substantial impact on potato improvement. Mapping studies in potato (at the 2x and 4x levels) have been conducted since the late 1980's [10-15], but marker-assisted selection (MAS) is not widely practiced in varietal breeding. To date, only a few molecular markers for economically important traits have been developed in potato, and most of these are for resistance to pests and diseases, including late blight [16], Potato Virus Y [17-19], potato cyst nematode [20] and *Verticillium *wilt [21]. Development of a genome-wide set of markers polymorphic in elite germplasm would allow more cultivars and breeding clones to be genotyped and substantially advance potato breeding.

With the emergence of genomics in the late 1990s, Expressed Sequence Tag (EST) projects were initiated for potato in which Sanger-based sequencing was used to catalog transcripts in an array of tissues and genotypes [22-26]. To date, 237,583 sequences derived by Sanger sequencing are available for potato in the National Center for Biotechnology Information (NCBI) dbEST (Release 011110;[27]). While prior sequencing has provided a useful starting point for detecting polymorphic loci in potato, the polymorphisms that can be defined at present are restricted to the genotypes sequenced to date and the depth of sequencing performed. Three cultivars, 'Bintje' (1905), 'Kennebec' (released in 1948), and 'Shepody' (1980), have substantial Sanger sequence datasets, and for all three cultivars, relatively low-coverage Sanger sequencing was employed.

Due to the high throughput and low costs, next generation sequencing methods provide a powerful means to generate large sequence datasets that can be used to characterize sequence diversity [28,29]. In addition to discovery, next generation sequencing platforms can be used to rapidly generate polymorphisms and genotype data for genetic mapping [30-32]. To increase the number of single nucleotide polymorphisms (SNPs) available for basic and applied potato genetics, we conducted extensive transcriptome sequencing from three currently relevant potato cultivars, Atlantic [33], Premier Russet [34], and Snowden (released in 1990). Atlantic and Snowden are the two most widely grown public chipping cultivars in North America, while Premier Russet is a new, promising French fry clone. All three cultivars are used as parents in North American breeding programs. Using transcriptome data generated in this study, coupled with available Sanger potato ESTs, we computationally identified a large collection of SNPs for use in genotyping. We also created a germplasm panel of ^~^250 potato clones, which includes many representatives of each of the major market classes, *Solanum *species, genetic stocks, and represents a broad genetic base to assess the allelic distribution of a subset of SNPs and the population structure and relationships between market classes.

## Results and Discussion

### Sequencing and annotation of the potato transcriptome

The genotypes and sequence datasets used in this study are listed in Table 1. Using normalized cDNA libraries and the Illumina Genome Analyzer 2 (GA2) platform, we generated 7.0 Gb of sequence increasing by 60-fold the amount of transcriptome sequence available for potato (Table 2). To reduce the redundancy in both the Sanger and GA2 derived transcript sequences, we performed *de novo *assembly of quality-filtered reads. For the GA2 transcript data, *de novo *assembly resulted in a combined total of 86.9 Mb of contigs for potato (Table 2). Singletons (unassembled single reads) from the GA2 platform were not used in downstream bioinformatic analyses due to quality issues associated with single pass short reads. For the three accessions sequenced using the GA2 platform, a similar number of reads were available for assembly (36-40 million) and the assembled transcriptome size ranged from 28.6 to 29.4 Mb (Table 2). The narrow range of assembled transcriptome sizes within the potato GA2-generated datasets suggests that the underlying cDNA populations and the sequencing and assembly process were similar within the potato samples.

**Table 1:** Genotypes and sequence datasets used in this study,

Species	Cultivar	Market Class	Platform	Comments
*S.tuberosum *Group Tuberosum	Kennebec	Fresh market	Sanger ESTs	1948 release
*S.tuberosum *Group Tuberosum	Bintje	Fresh market	Sanger ESTs	1905 release
*S.tuberosum *Group Tuberosum	Shepody	French fry processing	Sanger ESTs	1980 release
*S.tuberosum *Group Tuberosum	PremierRusset	French fry processing	GA2 ESTs	2008 release
*S.tuberosum *Group Tuberosum	Snowden	Chip processing	GA2 ESTs	1990 release
*S.tuberosum *Group Tuberosum	Atlantic	Chip processing	GA2 ESTs	1978 release
*S.tuberosum *Group Phureja	DM	Diploid Andean Fresh Market	NA	Used in Genome Project^a^

^a^The DM genome is available at http://potatogenome.net.

**Table 2:** Potato sequence and assembly statistics.

	Sanger			GA2		
	Bintje	Kennebec	Shepody	Atlantic	Snowden	Premier Russet
Total No. sequences	15,866	83,549	86,341	36,291,638	38,981,546	39,556,178
Total No. Gb sequences	0.0079	0.0544	0.0543	2.2	2.4	2.4
						
No. sequences passed quality filters	14,588	78,386	83,611	30,185,186	31,949,096	33,288,120
No. of Gb of sequences passed quality filters	0.0077	0.0533	0.053	1.8	2.0	2.0
						
Total No. contigs & singletons	7,510	25,330	51,459	NA	NA	NA
No.contigs	2,332	10,318	10,716	45,214	58,754	54,917
No.singletons	5,178	15,012	40,743	NA	NA	NA
						
Total No. Mb contigs & singletons	4.27	19.89	36.33	29.45	28.55	28.93
No. Mb contigs	1.61	10.6	8.68	29.45	28.55	28.93
No. Mb singletons	2.66	9.29	27.65	NA	NA	NA
						
N50 contig size (bp)	711	1,097	847	1,192	775	826
Max contig size (bp)	2,255	4,081	2,517	11,317	7,012	6,675
Min contig size (bp)	278	272	847	150	150	150

**Figure 1:**
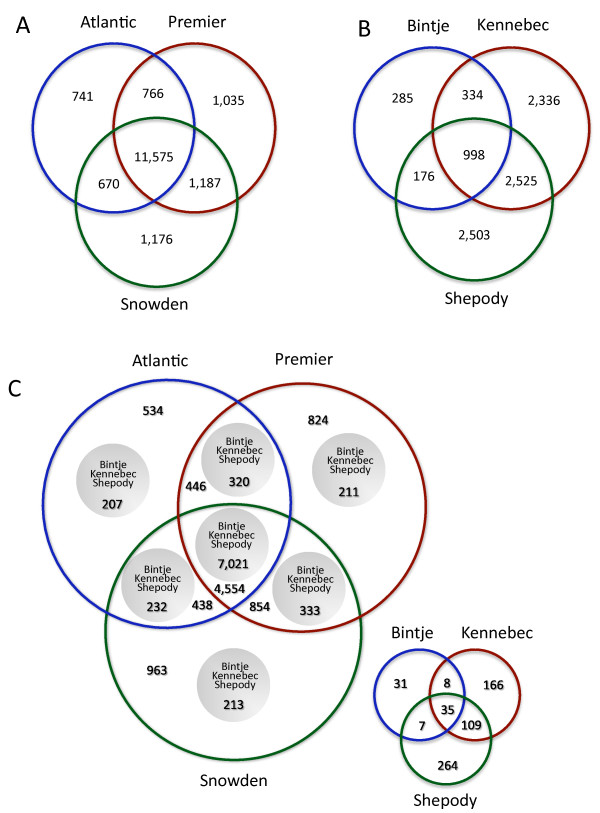
**Overlap of potato transcriptomes**. Contigs from each of the cultivars were searched against the *A. thaliana *proteome and the non-redundant *A. thaliana *proteome matches are shown. A. GA2-generated transcript datasets; B. Sanger-generated transcript datasets; C. Nested Venn diagram with all six datasets. The small Venn diagram within C shows the overlap between contigs found only within the Sanger datasets.

Although we generated a significant amount of non-redundant transcriptome sequence via our GA2 platform datasets, this does not represent the complete transcriptome as it is unlikely that all genes were expressed in the tissue samples used for cDNA synthesis, transcripts expressed at a low level may have been missed in our sampling, and coverage of the transcript may be insufficient to yield a full length assembly. To assess the representation of the potato transcriptome, we aligned the contigs to the *Arabidopsis thaliana *proteome. For the three GA2-derived transcriptomes, a similar number of total, as well as distinct alignments with the Arabidopsis proteome were present with a substantial amount of overlap between the three GA2-transcriptomes (Table 3, Figure 1A). Analysis of molecular function gene ontology terms within the three GA2-transcriptomes indicate a similar representation of biological activity (Additional File 1).

**Table 3:** Alignment of contigs to the *A. thaliana *proteome.

Cultivar	No. contigs with alignment^a^	No.non-redudundant alignment^b^
Atlantic	27,934	13,752
		
Premier Russet	32,369	14,563
Snowden	33,503	14,608
		
Bintje	2,111	1,793
Kennebec	9,320	6,193
Shepody	9,163	6,202

^a^Contigs were search against the *A. thaliana *proteome using an E value cutoff of <10^-5^. Only the top alignment was retained.

^b^Multiple alignments to the same *A. thaliana *protein were condensed to provide a non-redundant estimation of representation of the *A. thaliana *proteome.

We compared our GA2-generated assemblies to EST collections generated previously using the Sanger platform [22-25]. The three Sanger EST datasets (Bintje, Kennebec, and Shepody) were more variable in number of reads: 15,866, 83,549, and 86,341, respectively, and consequently, the Sanger-derived assemblies were more variable in representation of the potato transcriptome: Bintje (4.3 Mb), Kennebec (19.9 Mb), Shepody (36.3 Mb)(Table 2). Due to smaller sampling of the transcriptome, Bintje was under-represented compared to Kennebec and Shepody as shown by the reduced number of total and non-redundant alignments to the Arabidopsis proteome compared to the GA2-generated transcriptomes (Table 3). When examined for overlap based on alignment to the Arabidopsis proteome (Figure 1B), these three datasets do overlap with each other, although the skew in total numbers of contigs between the three cultivars is reflected in overlap of non-redundant Arabidopsis alignments. The vast majority (>90%) of the Sanger-generated contigs were represented within the GA2 datasets (Figure 1C).

### SNP discovery

**Figure 2:**
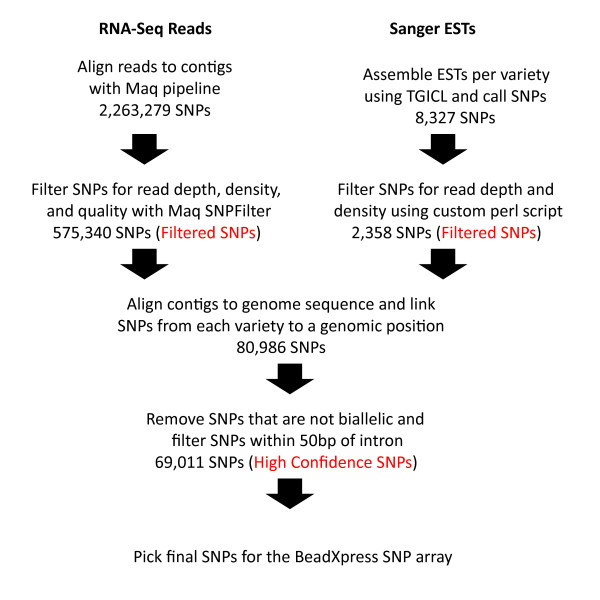
**Workflow used for SNP discovery in potato transcriptomes and design of the BeadXpress SNP array**. SNPs identified in RNA-Seq reads were called and filtered using the Maq SNP pipeline. Sanger ESTs were clustered by cultivar using TGICL [40] and SNPs called and filtered using custom Perl scripts. Filtered SNPs were linked to positions of the potato DM genomic sequence and filtered again to eliminate those close to an intron as well SNPs that were not biallelic. SNPs selected for the BeadXpress SNP array were selected randomly from the Atlantic, Premier Russet, and Snowden datasets.

SNPs were abundant within and between the transcriptomes. At the first stage of the SNP discovery pipeline with limited filtering, 2,263,279 SNPs were called by Maq in the GA2-generated transcriptomes. Application of read depth, density, and quality score filters with the Maq SNPFilter reduced the SNP count among the three GA2-derived transcriptomes to 575,340 SNPs (i.e., Filtered SNPs; Figure 2). In parallel, with three Sanger-derived transcriptomes we identified 2,358 Filtered SNPs. As these SNPs were identified on transcript assemblies, there is overlap between the SNPs in the six cultivars. Thus, we used the potato draft genome sequence available from the Potato Genome Sequencing Consortium [35] to align the contigs from all six cultivars and identify redundant SNPs resulting in 80,986 unique SNPs among all six cultivars. Using a stringent set of filters to address intron/exon boundaries, paralogs, non-biallelic SNPs and Illumina design specifications, we were able to identify 69,011 high confidence SNPs from Atlantic, Bintje, Kennebec, Premier Russet, Shepody, and Snowden that could be used on a SNP genotyping platform (Additional File 2). It should be noted that there are additional high quality SNPs outside of the 69,011 SNPs and that this reduced dataset was created solely for generation of an Illumina SNP genotyping platform.

From these 69,011 SNPs, 96 were empirically tested using the Illumina BeadXpress genotyping platform, of which 82 were considered high quality (Additional File 3). Due to the partial nature of transcriptome sequence due to expression levels and sequencing depth, full coverage of each SNP for all three genotypes (Atlantic, Premier Russet, and Snowden) was not available. RNA-seq based genotypes were available for all three genotypes for 14 SNPs, two genotypes for 20 SNPs, and one genotype for 48 SNPs. Of the 82 high quality SNPs, 70 were congruent for all genotypes between the two platforms, 10 were inconsistent for one of the genotypes, and two of the SNPs failed for one of the alleles in the BeadXpress assay or are homozygous for the genotypes used in this study. Thus, our computational pipeline to predict SNPs solely from sequence data is robust. In total, from the 182,251 Sanger and GA2-generated contigs, 82,780 contigs have at least one high confidence SNP. The remaining 99,471 contigs lack a high confidence SNP that meets our filtering criteria.

### Germplasm population structure

**Figure 3:**
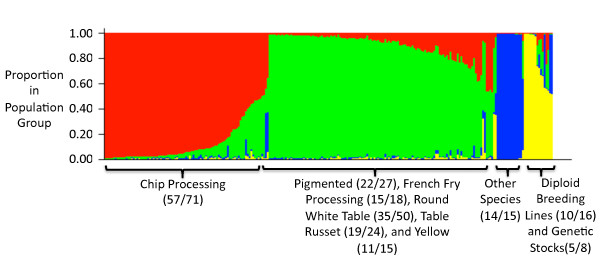
**Graphical display of population substructure for 248 genotypes at a population size *K *= 4**. Population substructure was determined using STRUCTURE [47] with 82 high quality SNP markers. Each genotype is represented by a vertical line. Color segments within the vertical line indicate the proportion of membership in each of the four population substructure groups. Population substructure groups are color-coded as population one (red), population two (green), population three (blue), and population four (yellow). Numbers in parenthesis indicate the number of genotypes with majority membership (greater than 50%) in each population group and the total number of genotypes for each market class.

Intra- and intervarietal SNP diversity exists in elite cultivated germplasm. With the diversity of a germplasm panel and the availability of a set of random SNP markers, the opportunity exists to examine population structure in elite breeding germplasm. US potato breeding efforts currently center on improving six distinct market classes (chip processing, French fry processing, pigmented, table russet, round white table, yellow), where most (but not all) hybridizations for varietal selection are made between clones within a market class. Over time one might expect these six market classes to diverge, not only in terms of the few traits that define each class, but also in terms of unlinked, selectively neutral DNA markers. To assess whether the market classes have diverged significantly, 82 high quality SNPs (selected from the 96 SNPs used in BeadXpress genotyping described above) were used to evaluate the 248 clone germplasm panel. Using the likelihood of the observed genotypes given the number of populations in the model for each of the values of *K *tested (*K *= 2-10), it was determined that the number of subpopulations in the set of 248 diverse genotypes was four (Additional File 4). By pedigree, these four groups are comprised predominantly of 1) chip processing germplasm, 2) all other tetraploid market classes combined (pigmented, French fry processing, round white table, table russet, and yellow), 3) wild (non-*S. tuberosum*) species, and 4) diploid breeding lines derived from various *Solanum *species and genetic stocks (Figure 3 and Additional File 5). It is not surprising that the wild species group is distinct from *S. tuberosum *germplasm. The 2× breeding lines and 4x *S. tuberosum *germplasm grouped separately despite our inability to score allele dosage in 4x germplasm (i.e., we were not able to differentiate AAAB, AABB, and ABBB in tetraploids - all were scored as AB). We were initially surprised that the cultivated tetraploid germplasm differentiated into two groups based on market class (chip processing versus all other market classes), rather than one group (containing all market classes) or six groups (one for each market class). Over the past fifty years, chip processing germplasm has undergone intense selection for processing characteristics such as low reducing sugar content, high starch, shallow eyes and round tuber appearance, which may partially explain why this market class is genetically distinct from all other market classes.

**Figure 4:**
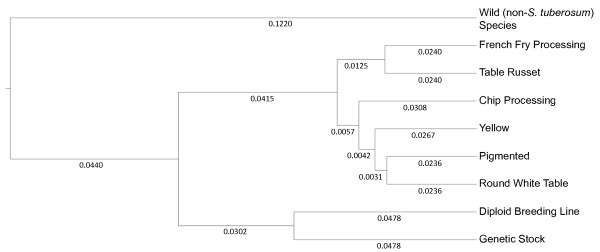
**Unweighted Pair Group Method with Arithmetic Mean (UPGMA) tree of 244 genotypes categorized by market class based on 82 high quality SNP markers**. The numbers above each branch are the branch length, which relates to the genetic distance between groups.

Of the 248 potato clones genotyped, 244 could be readily categorized as either wild species, diploid breeding line/genetic stock, or as belonging to one of the following market classes: French fry processing, table russet, chip processing, yellow flesh, pigmented skin, round white table. Using allele frequency-based distances (Additional File 6), the genetic similarities between individual market classes, as well as more distant germplasm, were determined for the 244 genotypes that could be categorized (Figure 4). Similar to what was observed in the population structure analysis, the wild species group is distinct from all *S. tuberosum *germplasm. The wild species group thus serves as an outgroup for analysis of elite potato germplasm. The diploid breeding line/genetic stock group clustered more closely to the cultivated germplasm, presumably because members of this group often contain substantial amounts of *S. tuberosum *Group Tuberosum and/or Phureja. Even so, the diploid breeding/genetic stock group was still clearly distinct from cultivated germplasm. Interestingly, within cultivated germplasm the two russet market classes - French fry processing and table russet - clustered more closely to each other than they did to either the round white chip processing and round white table groups. We had expected the French fry processing and chip processing market classes to group more closely because of similar selection for processing traits. Russet germplasm, common in North America but not elsewhere, is characterized by long tuber shape and russet skin. The group with red or purple skin formed its own cluster, while the remaining cluster included various round tuber classes (round white table, round white chip processing, yellow flesh). The yellow flesh, pigmented skin, and round white table groups clustered separately from the chip processing group.

### Computational SNP analysis across six sequenced cultivars

While the BeadXpress assay allowed us to examine 82 SNPs across 248 germplasm clones, it is also possible to compare a much larger number of SNPs across the six potato cultivars for which there is available transcriptome sequence. Using a separate computational pipeline that mapped sequence reads directly to the DM reference genome [35], we identified 2,117,754 raw SNP calls, of which, 147,525 SNPs remained after filtering with the Bowtie/SAMTools pipeline. Collectively, these mapped to 101,487 unique genome positions and represented approximately 25% more SNPs than the Maq pipeline described above, which was based on transcript assemblies.

When comparing heterozygous autotetraploid potato cultivars, SNPs can be intra- or inter-varietal. As shown in Table 4, SNPs were readily detected within all six accessions, ranging from 1,155 in Bintje to 46,074 in Premier Russet, reflective of the increased transcriptome data available for Premier Russet compared to Bintje. For all six accessions examined, regardless of platform, approximately half of the SNPs detected in each transcriptome were restricted to that cultivar (Table 4). This may reflect true exclusivity of the SNPs, a lack of sampling depth in the other five transcriptomes sufficient to permit SNP detection under our alignment and filtering criteria, and/or lack of expression of some alleles in some cultivars.

**Table 4:** Total and cultivar-restricted SNPs in six potato cultivars.

Cultivar	Total SNPs	Cultivar-Restricted SNPs
Atlantic	42,928	19,442
Snowden	46,074	21,559
PremierRusset	45,772	18,764
		
Bintje	1,155	576
Kennebec	8,773	5,533
Shepody	2,823	1,532

Total and cultivar-restricted SNPs were determined by aligning reads to the DM reference genome and calling SNPs using SAMTools. Cultivar-restricted SNPs are SNPs that are found only in a single cultivar with the other cultivars lacking the SNP or having no sequence data at that genomic position.

We determined the overlap of SNPs across all six cultivars in a pair-wise manner as well as the number of SNPs shared or restricted within market classes. Not surprisingly, the total number of SNPs in any pair-wise comparison was reflective of the initial size of the transcriptome and consequently, the number of SNPs identified (Table 5). The largest number of SNPs identified within a market class was for Snowden and Atlantic (17,531 SNPs), two chip processing cultivars. Of these, 7,570 SNPs were restricted to Snowden and Atlantic; some of these shared unique SNPs might have originated in Lenape, a parent shared by Atlantic and Snowden. For the French fry processing class, 535 SNPs were common to Premier Russet and Shepody, of which 106 were restricted to these two cultivars. For the fresh market class (Kennebec and Bintje), 329 SNPs were common with 141 restricted to these two cultivars. The 30-50 fold less SNPs common to the French fry and fresh market classes is attributable to the smaller datasets in the Sanger-generated transcriptomes (Bintje, Kennebec, Shepody). As we have not exhaustively sampled these six transcriptomes, especially those of Bintje, Kennebec, and Shepody, the SNP overlap reported reflects trends and not absolute numbers as we are under-estimating the inter-varietal SNPs. Thus, we expect the fraction of cultivar exclusive SNPs to be reduced as more cultivars are sequenced.

**Table 5:** Pairwise comparison of SNPs between potato accessions.

Cultivar 1	Cultivar 2	Total SNPs	Cultivar-restricted SNPs
Atlantic	Premier Russet	14,955	5,087
Atlantic	Snowden	17,531	7,570
Atlantic	Bintje	192	40
Atlantic	Shepody	506	128
Atlantic	Kennebec	1,459	388
Premier Russet	Snowden	18,537	8,365
Premier Russet	Bintje	212	42
Premier Russet	Shepody	535	106
Premier Russet	Kennebec	1,689	424
Snowden	Bintje	215	31
Snowden	Shepody	567	121
Snowden	Kennebec	1,665	349
Bintje	Shepody	136	31
Bintje	Kennebec	329	141
Shepody	Kennebec	566	276

Pairwise comparision of SNPs across the six potato cultivars. Cultivar-restricted SNPs are SNPs found exclusively in the two cultivars based on alignment to the reference genome.

## Conclusions

By combining RNA-Seq of three current cultivars (Atlantic, Premier Russet, Snowden) with data mining of existing ESTs from three older cultivars (Bintje, Kennebec, Shepody), we were able to identify an abundance of SNPs in elite potato germplasm. These SNPs will facilitate future marker analyses by potato geneticists and breeders alike. Breeders, in particular, will soon be able to incorporate large amounts of genotypic data into their decision making. This will lead to deeper understanding of breeding germplasm, as well as more efficient QTL mapping, association mapping and marker-assisted selection, collectively resulting in more predictable and directed breeding.

With stringent filtering of sequence data in combination with alignment to a reference potato genome, we were able to identify 69,011 high confidence SNPs for use with the Infinium genotyping platform. A subset of these SNPs was recently used to design a 8300 marker SNP array [36]. The current study sought to validate 96 of these SNPs on 244 potato clones; 82 of these SNPs (85%) could be reliably scored. Genotyping with the validated 82 SNP markers allowed us to examine population structure and relationships between market classes. Even with this small number of SNPs, we were able to gain insight into the genetic structure of cultivated potato. Somewhat unexpectedly, we observed that chip processing germplasm is discernibly different from other market classes, even though intense selection for chip processing traits is a relatively recent phenomenon, only practiced for the past 50 years or so, and in a crop where meioses are relatively infrequent. We also found that chip and French fry processing germplasm appear more closely related to round white table and table russet germplasm, respectively, even though the traits required for processing are similar across these two market classes.

## Materials and methods

### Germplasm and datasets used in this study

The germplasm panel was compiled from elite potato germplasm from 16 breeding programs across the U.S. including six international programs. Germplasm panel member names, market classes and species composition are noted in Additional File 5. Clones in the germplasm panel were assigned to market classes as follows. The long shaped potatoes were classified as table russet or French fry processing based upon their utilization. Similarly, the round white potatoes were classified as table or chip processing. The yellow market class is composed of yellow-fleshed clones, but does not include chip processing clones. The pigmented market class combines red and purple-skinned clones, some of which also have red or purple flesh. The diploid breeding lines consist of clones used by breeders for breeding or mapping purposes. The genetic stocks consist of clones used for genetic studies only; these clones have little or no value for breeding. To define the clone's genome composition, contributing breeders were asked to note if a potato clone contains wild species in its background, either as a parent or as a grandparent. A core set of *Solanum *species and accessions (provided by D. S. Spooner, USDA/ARS) that have previously been used for introgression into tetraploid germplasm were included in the panel to provide a taxonomic perspective. These clones were designated as "species" in our analyses. Sequences used in this study are listed in Table 1. Sanger ESTs from Bintje, Kennebec, and Shepody were obtained from NCBI dbEST [27]. Genomic sequences for *Solanum tuberosum *Group Phureja DM1-3 516R44 (DM) potato were obtained from the Potato Genome Sequencing Consortium ([35];v3 assembly).

### Transcriptome sequencing

RNA was isolated from young tuber meristems, leaves, flowers and callus of Atlantic, Premier Russet, and Snowden [37] and pooled in equimolar concentration. cDNA was synthesized and prepared for paired-end sequencing as described [38]. Samples were sheared, 300-350 bp fragments selected, and were normalized using double-stranded nuclease that digests high copy double-stranded DNA during re-association after denaturation. Each normalized library was sequenced in two paired-end (forward and reverse) lanes of 61 bp on the Illumina Genome Analyzer (Illumina Inc., San Diego, CA). Sequences are available in the SequenceRead Archive at NCBI (Study number SRP006384).

### *De novo *assembly and annotation of transcripts

Illumina RNA-Seq reads from each cultivar (Atlantic, Premier Russet, and Snowden) were assembled separately using the Velvet assembler [39] in the paired-end mode with a hash length of 31 and a minimum contig length of 150 bp. The insert size and expected coverage parameters were 350 bp and 31.2X for Atlantic, 300 and 34.4X for Premier Russet, and 300 bp and 33X for Snowden, respectively. Sanger-generated ESTs for Bintje, Kennebec, and Shepody were passed twice through SeqClean and assembled into contigs using the TGICL clustering pipeline [40].

The contigs (Velvet or TGICL-generated) were searched against the *A. thaliana *proteome (TAIR9; [41]) and UniRef100 [42] using BLASTX [43] with an E-value cutoff of 1e-5. To annotate the potato contigs, the first meaningful functional annotation was selected from the top 10 scoring BLAST matches to the UniRef100 database and transitively assigned to the potato contig. If no meaningful annotation was found in the top 10 UniRef100 matches yet there was a match meeting the cutoff criterion, the potato contig was annotated as a "conserved gene of unknown function". If no hits at all were found within the cutoff criterion, the potato contig was annotated as a "gene of unknown function". For representation of the Arabidopsis proteome, contigs were searched against *A. thaliana *proteome (TAIR9; [41]) with an E-value cutoff criterion of 1e-5 and the best alignment retained. For gene ontology associations, alignments to the *A. thaliana *proteome (TAIR9; [41]) were used to transitively assign gene ontology (GO; [44]) terms.

### SNP discovery and allelic diversity in a potato germplasm panel

We computationally identified SNPs within our three GA2-generated transcriptomes (Atlantic, Premier Russet, Snowden) and designed a 96 SNP BeadXpress assay to 1) validate our computational predictions and 2) assess allelic diversity and population structure in a diverse set of potato germplasm.

#### SNP selection

Intra-varietal and inter-varietal SNPs were identified by aligning the RNA-Seq reads from each variety to the Velvet-generated contigs using the Maq easyrun.pl pipeline in the paired-end mode (Figure 2; [45]). We imposed multiple sets of filters for the SNPs to be included in the BeadXpress assay. First, raw SNP calls from the pipeline were filtered with the maq.pl SNP filter script using a minimum depth of 20 reads, a maximum depth of 225, a minimum consensus score of 30, a minimum adjacent consensus score of 20, and a required maximum mapping quality of 60. Additional constraints were a maximum of one other SNP in a 100 bp flanking window and that the SNP must be located 50 bp from areas identified as indels by the pipeline [maq.pl SNPfilter -d 20 -n 20 -Q 60 -q 30 -w 50 -N 2 -W100 -f cns.indelse -F cns.indelpe cns.snp]. Second, the SNPs were filtered to exclude SNPs near intron-exon junctions by aligning the Velvet contigs to the DM scaffolds [35] using GMAP [46]. Only SNPs located within exons that aligned at >95% identity with no gaps were retained while SNPs within 50 bp of an exon-intron boundary were discarded. Third, only biallelic SNPs were retained. Fourth, remaining SNPs were scored by Illumina (San Diego, CA) for suitability for the Infinium BeadXpress platform and SNPs with a score <0.9 or a fail code were discarded. The final 96 SNPs selected for BeadXpress validation originated from Atlantic, Snowden, and Premier Russet (Additional File 3).

#### Genotyping potato germplasm

DNA was extracted from 248 potato lines using the Qiagen Qiaxtractor DX system (Qiagen Inc., Valencia, CA). Samples were loaded at 50 ng/μl on an Illumina BeadXpress Analyzer (Illumina inc., San Diego, CA) and data were analyzed using the Illumina GenomeStudio software. Cluster positions for three marker classes (AA, AB, and BB) were manually determined for each marker within the Illumina GenomeStudio software. Due to the difficulty of calling allelic dosage in the tetraploid clones, all heterozygous classes in tetraploids (AAAB, AABB and ABBB) were scored as AB. Of the 96 SNP markers, 14 were of low quality based on the tightness of clusters and/or signal intensity and were removed from downstream analysis (Additional File 3). Genotypic data for the remaining 82 high quality SNPs is provided (Additional File 7). Population structure was determined using the STRUCTURE software [47]. Three iterations were run per *K *(number of populations) for *K *equals two through 10 using an admixture model with a burn-in time and replication number of 50,000. The population number with the maximum likelihood of the observed genotypes given the number of populations was used to determine population structure. PowerMarker version 3.25 [48] was used to calculate the allele frequency based genetic distance between the market classes using the Rogers distance method [49] for the 244 genotypes with defined market classes. An unweighted pair group method with arithmetic mean (UPGMA) tree was constructed based on the Rogers distances; FigTree version 1.3.1 was used to produce the UPGMA tree image [50].

### Cross-comparative analyses of SNPs across six cultivars

Access to large transcriptome sets for six potato cultivars provides an opportunity to examine allelic diversity across a wide range of loci, albeit from a limited set of germplasm. To compare SNPs across the Atlantic, Bintje, Kennebec, Premier Russet, Shepody, and Snowden transcriptomes, we used a computational approach modified from that described above. Instead of aligning transcripts with each other, reads were directly mapped to the genome.

#### Illumina transcript datasets (Atlantic, Premier Russet, Snowden)

RNA-Seq reads from Atlantic, Premier Russet, and Snowden were mapped directly onto the DM reference genome sequence [35] with Bowtie (version 0.12.3; [51]). Only alignments of reads that mapped uniquely to the 15 genome were retained. The resulting SAM alignment file was processed using the SAMTools (version 0.1.7; [45]) package and initial SNP calls made (samtools pileup -vcf). The SNPs were then filtered with the samtools.pl varFilter script (samtools.pl varFilter -d 20 -D 240 -W 100 -N 2 -w 50) retaining SNPs with a minimum read depth of 20, a maximum read depth of 240, a minimum distance of 50 bp from putative insertions/deletions (indels), and only one other SNP within a 100 bp window around the SNP. Further filtering of the SNPs was done with a custom Perl script that removed SNPs with a consensus score <20, a SNP quality score <20, and a minimum mapping score of 60. As a final constraint, SNP calls that had greater than 10% of 3' end of aligned reads were excluded to avoid calling alignment errors as SNPs. The genomic positions of the SNPs and associated metadata were stored in a PostgreSQL relational database using the Chado schema [52].

#### Sanger transcript datasets (Bintje, Kennebec, Shepody)

For Sanger-generated sequences, SNPs were called using a custom Perl SNP-calling script that required an overall read depth of 10, of which, 4 reads had to support the SNP call. The SNP calls were then filtered removing SNPs with 50 bp of an intron and SNPs with more than one additional SNP in a 100 bp window surrounding the SNP.

## Authors' contributions

JPH, CNH, BRW, ANM analyzed data. KS and AVD generated data. AVD WSdJ, DSD, and CRB oversaw the experiments. All authors participated in writing the manuscript and approved the final manuscript.

## Supplementary Material

Additional file 1 **Functional classification of potato transcriptomes**. The distribution of annotated contigs from each of the cultivars, Atlantic (A), Premier (B), and Snowden (C) based on their annotations to terms in the gene ontology molecular function category are shown. Click here for file

Additional file 2 **High confidence SNPs for genotyping**. This file lists the high confidence SNPs with their flanking sequence that were used in the genotyping experiment. Click here for file

Additional file 3 **Genes and SNPs represented on the 96 BeadXpress platform**. This file lists the SNPs and the genes represented by the SNP with their functional annotation. Click here for file

Additional file 4 **Graphical display of population substructure for 248 genotypes at variable population numbers (*K *= 2, 3, 4, 5, 6, 7, 8, 9, and 10)**. Population substructure was determined using STRUCTURE [47] with 82 high quality SNP markers. The number of populations with the maximum likelihood of the observed genotypes given the number of populations is indicated by *. Click here for file

Additional file 5 **Germplasm genotyped with the BeadXpress SNP assay**. Population structure was determined using STRUCTURE [47] with 82 high quality SNP markers. Clones used in the genotyping experiment, their species composition, market class, and their proportion in the groups are shown. Click here for file

Additional file 6 **Rogers genetic dissimilarity matrix **[49]**between market classes based on 82 high quality SNP markers and 244 genotypes with known market class designations**. Genetic dissimilarity among market classes are shown. Click here for file

Additional file 7 **Genotypic data for 82 high quality BeadXpress SNP makers on 248 genotypes**. Genotypes for potato clones with 82 SNP markers are shown. Click here for file
