# Supplementary figures and images for: Single nucleotide polymorphism discovery in elite north american potato germplasm
Source: BMC Genomics. 2011 Jun 9;12:302. doi: 10.1186/1471-2164-12-302 (PMC3128068; doi:10.1186/1471-2164-12-302)

**A**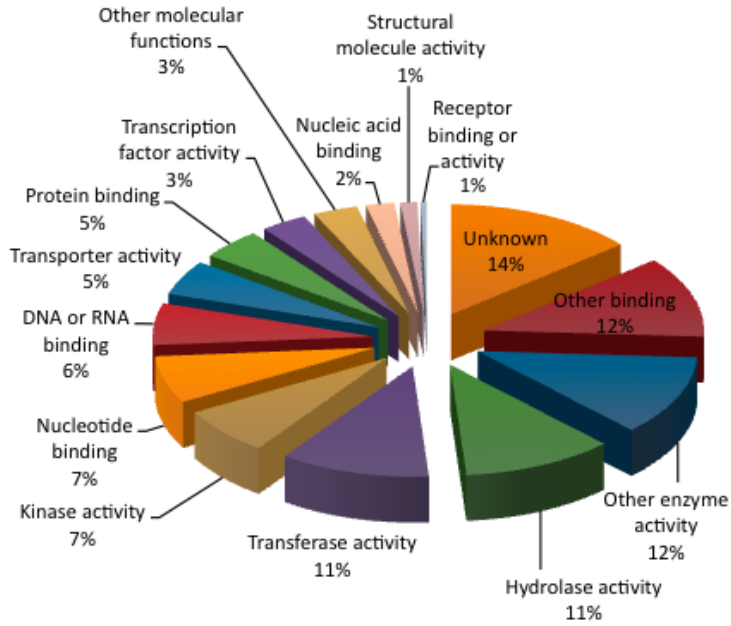**B**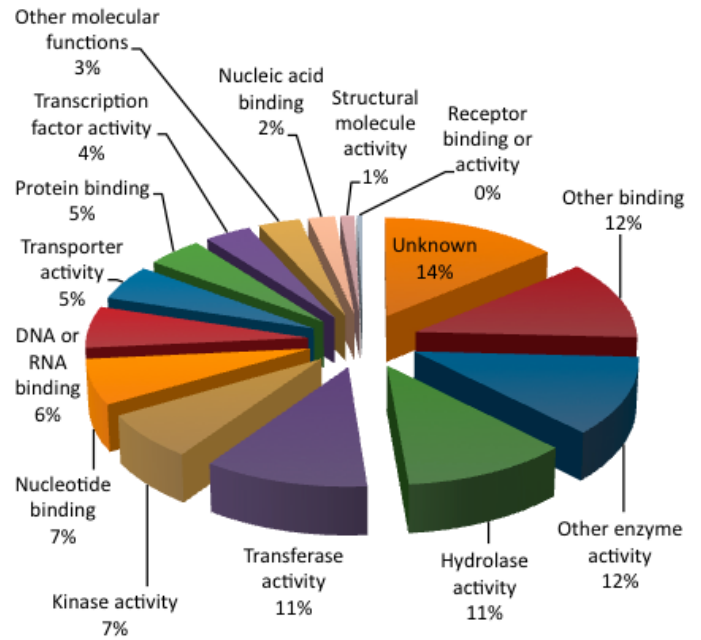**C**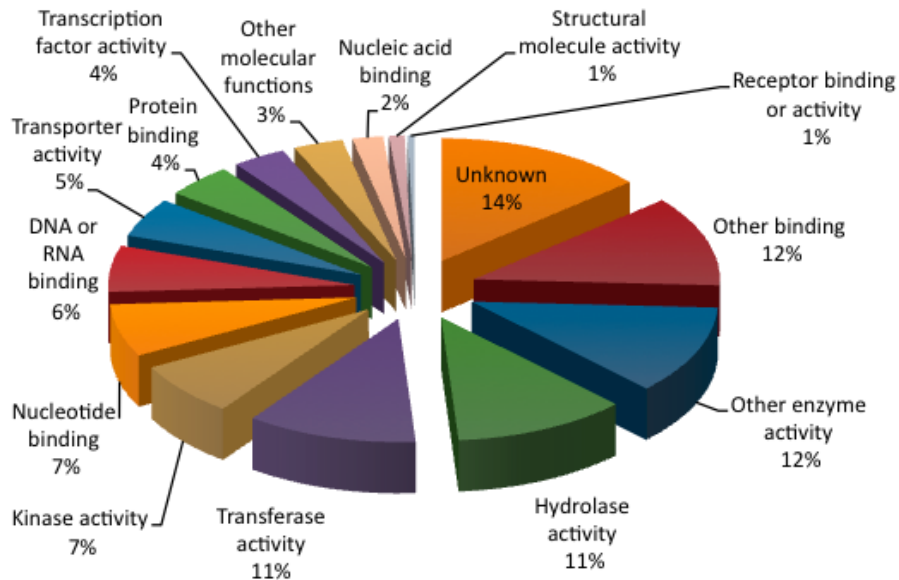

Supplement: Additional file 1 — Functional classification of potato transcriptomes. The distribution of annotated contigs from each of the cultivars, Atlantic (A), Premier (B), and Snowden (C) based on their annotations to terms in the gene ontology molecular function category are shown. [file 1471-2164-12-302-S1.PDF]

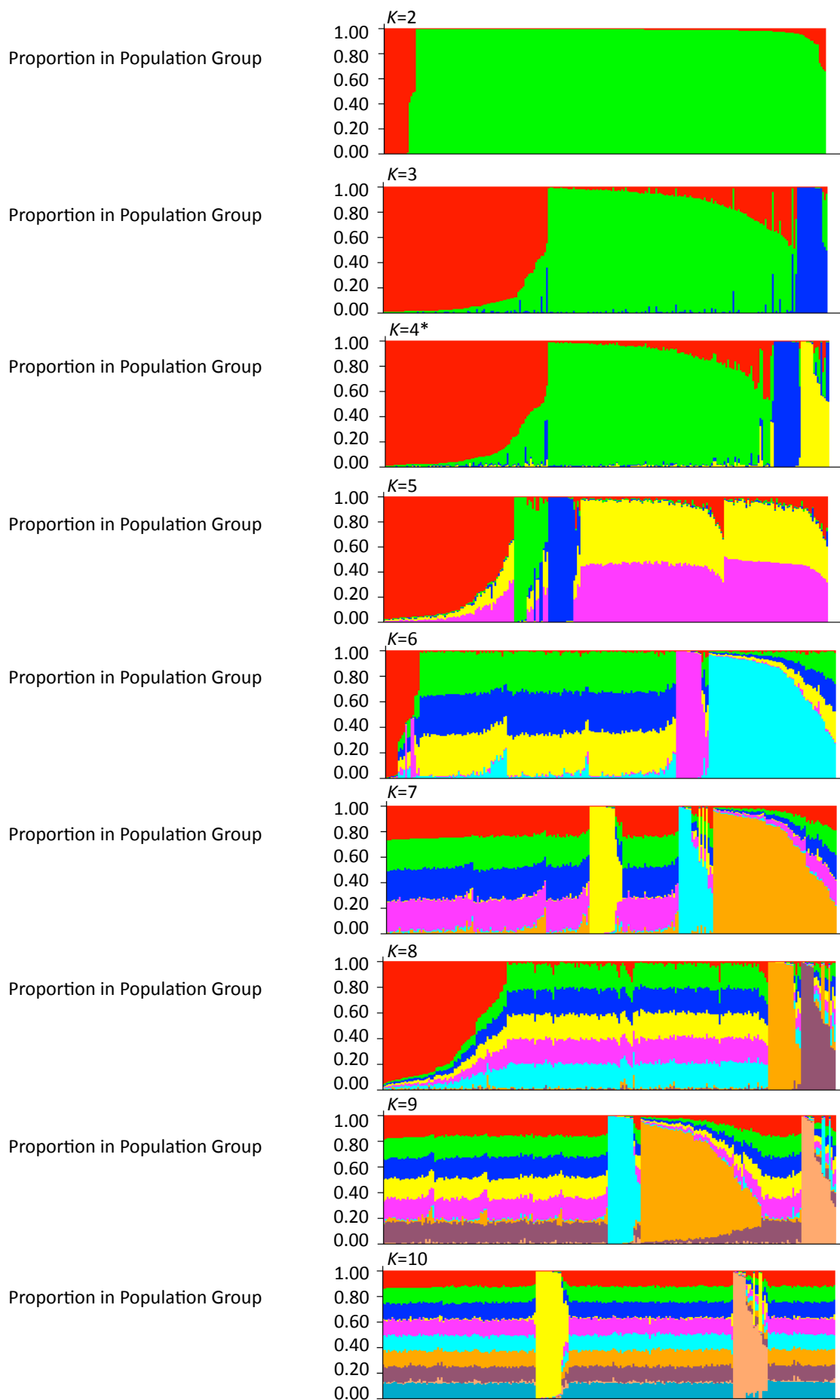

Supplement: Additional file 4 — Graphical display of population substructure for 248 genotypes at variable population numbers (K = 2, 3, 4, 5, 6, 7, 8, 9, and 10). Population substructure was determined using STRUCTURE [47] with 82 high quality SNP markers. The number of populations with the maximum likelihood of the observed genotypes given the number of populations is indicated by *. [file 1471-2164-12-302-S4.PDF]
